# Shaoyao-Gancao Decoction Relieves Visceral Hyperalgesia in TNBS-Induced Postinflammatory Irritable Bowel Syndrome via Inactivating Transient Receptor Potential Vanilloid Type 1 and Reducing Serotonin Synthesis

**DOI:** 10.1155/2020/7830280

**Published:** 2020-10-15

**Authors:** Yun-Yun Shao, Yi-Ting Guo, Jian-ping Gao, Jun-Jin Liu, Zhuang-Peng Chang, Xiao-Juan Feng, Ding Xu, Gui-Feng Deng, Rui-Gang Hou

**Affiliations:** ^1^School of Pharmaceutical, Shanxi Medical University, Taiyuan, Shanxi 030000, China; ^2^Department of Pharmacy, Second Hospital of Shanxi Medical University, Taiyuan, Shanxi 030000, China

## Abstract

Postinflammatory irritable bowel syndrome (PI-IBS) is a common functional gastrointestinal disorder, which is characterized by abdominal pain, low-grade inflammation, and visceral hypersensitivity. Shaoyao-Gancao decoction (SGD) has been used to improve the clinical symptoms of abdominal spasmodic pain accompanying acute gastroenteritis, but the underlying therapeutic mechanism has not been fully elucidated. In the present study, a rat model of PI-IBS was established via rectal administration of TNBS. Rats were scored daily for 28 days using disease activity index (DAI). Abdominal withdrawal reflex (AWR) was used to measure the pain threshold. After SGD (6.25, 12.5, and 25 g/kg/d) treatment for 14 days, rat colonic tissue was collected for histopathological grading, enterochromaffin (EC) cell count, and 5-HT content measurement. RT-qPCR and western blot analyses were employed to detect the gene and protein level of tryptophan hydroxylase (TPH), serotonin reuptake transporter (SERT), and transient receptor potential vanilloid 1 (TRPV1). To further validate the effect of SGD on TRPV1, another experiment was performed in cells. The results revealed that visceral hyperalgesia, reflected by increased DAI, AWR, pathological injury score, 5-HT content, and EC cell count in PI-IBS rats, was significantly ameliorated by SGD. In cells, SGD markedly inhibited the expression and function of TRPV1. Moreover, the expression levels of TPH were also repressed by SGD. The findings of the present study indicated that the therapeutic effect of SGD on visceral hyperalgesia may be closely associated with the regulatory role of TRPV1 and 5-HT signaling pathways.

## 1. Background

As one of the most common functional gastrointestinal disorders, irritable bowel syndrome (IBS) is characterized by abdominal pain and altered bowel habits, with a prevalence of 10–20% in the general population [1]. Multiple triggering events are involved in IBS pathophysiology, including psychosocial disturbance, low-grade inflammation, visceral hypersensitivity, and altered microbiota, but the precise mechanisms remain elusive [2]. It has been reported that ∼7–31% of IBS patients develop the symptoms after an infectious event, and this subtype of IBS has been defined as postinflammatory IBS (PI-IBS). Patients with PI-IBS have more evident visceral hypersensitivity compared with IBS [3, 4], which may severely disrupt their daily life.

Visceral hypersensitivity is considered as the common clinical feature among all PI-IBS patients [5]. Increased sensitivity to distension of the colon and rectal ballooning are observed in ∼50% of PI-IBS patients. Although the underlying mechanisms remain unclear, multiple evidence indicates that a variety of neuron-localized ion channels, such as transient receptor potential (TRP), serotonin (5-HT), and gut mucosal mast cells and their mediator, histamine, play key roles in the process of PI-IBS [6]. Among the novel targets in the pain field, the transient receptor potential vanilloid 1 (TRPV1) has been attracting increasing attention [7]. It has been reported that the expression and function of TRPV1 are enhanced in preclinical models of visceral hypersensitivity, while clinical data suggest that TRPV1 antagonists may decrease the visceral hypersensitivity to colorectal distention, emphasizing the key role of TRPV1 in the pathological process of PI-IBS [8, 9].

5-HT is synthesized from tryptophan by tryptophan hydroxylase (TPH) in enterochromaffin (EC) cells and aromatic amino acid decarboxylase [10]. The 5-HT released from EC cells may activate serotonergic receptors and be taken up into the enterocytes by serotonin reuptake transporter (SERT). It was reported that increased EC cell numbers and elevated 5-HT bioavailability contribute to abdominal pain in IBS patients [11]. Therapeutic agents targeting various 5-HT receptor subtypes have been used in the treatment of motility disorders. For example, 5-HT3 receptor antagonists were used to treat postoperative nausea and vomiting, whereas 5-HT4 receptor agonists were administered to treat gastric emptying and constipation [11]. Moreover, 5-HT pathway also plays key roles in TRPV1 activation-induced visceral hypersensitivity. Thus, 5-HT signaling may represent a potential therapeutic target in PI-IBS.

According to traditional Chinese medicine (TCM), the pathogenesis of IBS is closely associated with Pi xu (insufficiency of the spleen) and liver depression [12]. Therefore, strengthening the spleen and soothing the liver is considered as a therapeutic strategy in the treatment of IBS. Shaoyao-Gancao decoction (SGD), also known as Shakuyaku-Kanzo-to in Japan, is a traditional Chinese herbal prescription composed of *Radix Paeoniae Alba* (*Paeonialactiflora* Pall. root; PA) and *Glycyrrhiza uralensis* (*Glycyrrhiza uralensis* Fisch., root and rhizome, honeyed; GU) at a ratio of 1 : 1 [13–15]. SGD exhibited excellent therapeutic efficacy in strengthening the spleen and soothing the liver [16–18]. Previous studies also demonstrated that SGD possesses analgesic and anti-inflammatory properties, and its effect may be derived from its bioactive components paeoniflorin and glycyrrhizin; thus, SGD is frequently used to improve the clinical symptoms of abdominal spasmodic pain accompanying acute gastroenteritis [19]. However, whether SGD exerts therapeutic effects on a PI-IBS experimental model and whether the underlying mechanism is relevant to the TRPV1 and 5-HT signaling pathways require further investigation. Thus, the aim of the present research was to investigate the therapeutic effect of SGD and its regulatory role on the TRPV1 and 5-HT signaling pathways.

## 2. Materials and Methods

2,4,6-trinitrobenzenesulfonic acid (TNBS) was purchased from Sigma-Aldrich; Merck KGaA (St. Louis, MO, USA). *Radix Paeoniae Alba* (Anhui, China) and *Glycyrrhiza uralensis* (Shanxi, China) were purchased from China National Pharmaceutical Group Co., Ltd. (Taiyuan, Shanxi, China). The plants were identified by Professor Jing-ping Zhang (Department of Pharmacy, Second Hospital of Shanxi Medical University). Pinaverium bromide was obtained from Abbott Laboratories Ltd. (Bangkok, Thailand). The standard substance, including oxypaeoniflorin, albiflorin, paeoniflorin, liquiritin, isoliquiritin, glycyrrhizin, and glycyrrhetinic acid, were purchased from the Chengdu Institute of Biology (Chengdu, Sichuan, China). Hematoxylin and eosin were purchased from Solarbio Life Science (Beijing, China). 5-HT primary antibody was purchased from Sigma-Aldrich, Merck KGaA. Capsaicin was purchased from Chengdu Institute of Biology, and capsazepine was purchased from Dalian Meilun Biotechnology (Dalian, Liaoning, China).

### 2.1. Animals

A total of 60 male adult Sprague-Dawley rats (weighing 200–220 g, license no.: SCKX (Jing) 2014-0013) were purchased from the National Institutes for Food and Drug Control. The rats were housed in a standard environmental breeding room (ambient temperature 20–22°C, humidity 55–60%) and they were individually housed in cages on a 12 h–12 h alternating light-dark cycle. All rats had free access to water and food. All experimental procedures were performed in accordance with the recommendations of the National Institutes of Health Guidelines for Care and Use of Laboratory Animals. The experimental protocol was approved and monitored by the Committee on Use of Human and Animal Subjects in Teaching and Research of the Second Hospital of Shanxi Medical University (2015KS001).

### 2.2. Preparation and Quantity Control of SGD

SGD was prepared as previously described [13]. Briefly, PA and GU were mixed and macerated in sterile double-distilled water (1 : 10, w/v) for 1 h and then decocted twice for 1 h each time. The decocted solution was combined and concentrated to 2.12 g crude material per ml. This SGD solution was stored at 4°C until use and administered by gavage to the rats at different doses.

**Figure 1:**
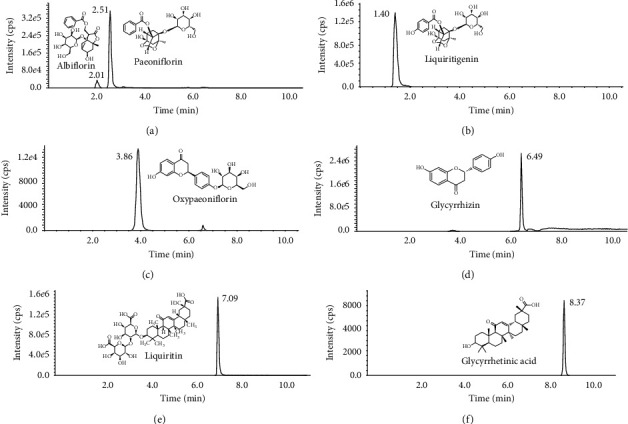
HPLC-QTRAP-MS/MS profiles to detect SGD. (a) Albiflorin and paeoniflorin, (b) liquiritigenin, (c) oxypaeoniflorin, (d) glycyrrhizin, (e) liquiritin, and (f) glycyrrhetinic acid.

In the present work, an ABI 5500 QTRAP mass spectrometer was used for quantity control of SGD. For UHPLC analysis, 2.12 g·mL^−1^ of SGD solution was dissolved in methanol : water. To determine the optimal ratio of methanol : water in which SGD is dissolved, different ratios were selected, such as methanol (100%), methanol : water 75 : 25 (v/v), methanol : water 50 : 50 (v/v), and methanol : water 25 : 75 (v/v). It was observed that the ratio 50 : 50 (v/v) had the best spectral response. Finally, a 250 ng·mL^−1^ dilution was selected for analysis of high content of ingredients in SGD. SGD was centrifuged at 16060 × g at 4°C for 30 min and then filtered and injected into an HPLC system for assay. The separation was performed using a KinetexXB-C18 column (2.1 × 100 mm, 2.6 *μ*m, Phenomenex, USA). In this study, the most abundant phytochemicals, including oxypaeoniflorin, albiflorin, paeoniflorin, liquiritin, isoliquiritin, glycyrrhizin, and glycyrrhetinic acid, were analyzed. The MRM chromatogram and quantitative analysis of the seven analytes in SGD are shown in Figure 1 and S1–S5.

### 2.3. TNBS-Induced PI-IBS Rat Model

The PI-IBS animal models were induced as previously described [20, 21]. Briefly, the animals were fasted overnight with free access to water, and a mixture solution of TNBS with 50% ethanol (0.8 mL per rat) was administered into the colon by a plastic catheter. The same volume of sterilized saline was administered to control rats. Following TNBS administration, DAI and pain pressure threshold of all the animals were measured.

### 2.4. Experimental Design

**Figure 2:**
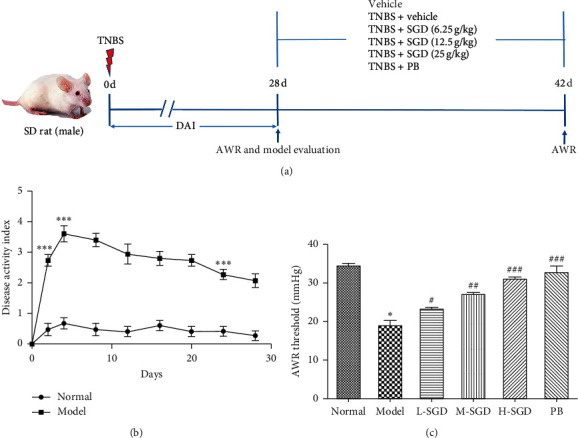
Evaluation of postinflammation irritable bowel syndrome (PI-IBS) model and the effect of SGD on alter visceral pain threshold in PI-IBS rats. (a) Experimental scheme of SGD on PI-IBS. (b) Disease activity index (DAI). (c) Abdominal withdrawal reflex test (AWR). Data are presented as mean ± SEM (*n* = 10). ^*∗*^*P* < 0.05 versus normal group; ^#^*P* < 0.05, ^##^*P* < 0.01, and ^###^*P* < 0.001 versus PI-IBS model rats.

The present study included *in vivo* and *in vitro* experiments. For the *in vivo* experiments, the 60 rats were randomly assigned to six groups (*n* = 10 per group) as follows: control group, PI-IBS group, and PI-IBS + SGD groups at doses of 6.25, 12.5, and 25 g^−1^·kg^−1^ d; and PI-IBS + pinaverium bromide (PB) group (Figure 2(a)). PB is commonly used to treat abdominal pain, as it blocks muscarinic receptors and calcium channels in intestinal smooth muscle cells [22, 23]. After SGD administration by gavage for two weeks, the pain threshold pressure test was performed. Subsequently, the rats were sacrificed and samples were collected for further analysis.

The *in vitro* experiment aimed to evaluate whether SGD could inhibit the expression of TRPV1 in HEK293 cells. HEK293 cells were divided into five groups as follows: control group; capsaicin (TRPV1 agonist); capsazepine (TRPV1 antagonist); SGD group; capsaicin + SGD group. After coculture for 24 h, the gene level of TRPV1 was detected by RT-qPCR analysis.

### 2.5. Disease Activity Index (DAI)

The DAI of each rat was evaluated daily for 28 days by a blinded observer as previously described [24]. Briefly, the rats were evaluated for body weight, stool consistency, and gross bleeding. These indicators were scored with 0–4 points, according to the rating criteria (Table 1), and the DAI scores were calculated by the average of the scores of these three indicators.

### 2.6. Abdominal Withdrawal Reflex (AWR)

The visceral pain threshold pressure was measured using the AWR test. Briefly, the rats were fasted for 12 h and anesthetized with ether, after which time a catheter with a balloon end was inserted into the colon. The rats were placed in clear plastic cages for regaining consciousness. After adapting for 30 min, CRD was applied with graded increments in its intensity until a visible contraction of the abdominal wall was observed. Each intensity level of distension was repeated five times with 10-minute intervals, and the average value from each animal was calculated for analysis.

### 2.7. Histological Staining

Following SGD treatment, the rats were sacrificed and colonic tissue was collected and washed in phosphate-buffered saline (PBS). Subsequently, colonic tissue was fixed in 4% formaldehyde buffer, cut into 4 µm sections, and stained with hematoxylin and eosin (H&E). The sections were observed under a light microscope for pathological grading. The pathological injury scoring criteria are presented in Table 2 [25].

### 2.8. Serotonin Content Assessment

A HPLC-MS/MS method was established to detect 5-HT content in the colonic tissue. First, iced trichloroacetic acid was used to homogenize the colonic tissue. After the supernatant was filtered and extracted with diethyl ether, derivatization solution was added to the samples. The 5-HT content was measured using high-performance liquid chromatography electrospray ionization tandem mass spectrometry (HPLC-MS/MS) with a Kinetex HILIC-column (2.1 × 100 mm, 2.6 *μ*m, Phenomenex, USA). The mobile phase was water/acetonitrile (25 : 75, v:v) and the total run time was 3 min. The flow rate was 0.3 mL·min^−1^ and the injection volume was 5 *μ*L. The peak areas were processed using the MultiQuant 3.0.2 software (AB SCIEX). The validation of the method was presented in the Supplementary Materials.

### 2.9. Immunohistochemistry and EC Cell Counting

Paraffin-embedded colon sections were deparaffinized and subjected to antigen retrieval. Then, the sections were incubated with 5-HT primary antibody (1 : 4,000, Absin Bioscience; catalog: abs120892). Subsequent visualization of the reaction product was performed using diaminobenzidine. For EC cell counting, five randomly selected fields at ×200 magnification were examined in each section using a microscope (Carl Zeiss Microscopy GmbH, Goettingen, Germany); the number of EC cells per mm^2^ of mucosa was quantified using Image J software (version 1.37C, US National Institutes of Health).

### 2.10. Cell Culture and Drug Treatment

*In vitro* experiment was performed to validate the effect of SGD on TRPV1. HEK293, a cell line with high expression of TRPV1, is widely used for transfection and can be easily induced by agonist [26, 27]. Therefore, in this study, capsaicin (TRPV1 agonist) or capsazepine (TRPV1 antagonist) were used to induce or inhibit the expression of TRPV1 in HEK293 cells, respectively. HEK293 cells (Fu Heng Biology Technology Co. Shanghai, China) were cultured in high-glucose DMEM with 10% FBS (GIBCO, USA). Cells were cultured at 37°C under 5% CO_2_ in humidified air for two to three days. Cell viability was detected by microplate reader with Cell Counting Kit-8 (CCK-8) assay (Boster, Wuhan, China). HEK293 cells were plated into 6-well plate at a density of 2.5 × 10^5^ cells per well. Capsaicin (TRPV1 agonist, 10 *μ*M, Chengdu Must Bio-Technology, Chengdu, China), capsazepine (TRPV1 antagonist, 10 *μ*M, Dalian Meilun Biotechnology, Dalian, China), SGD (100 *μ*g/mL), and capsaicin + SGD (10 *μ*M capsaicin for 1 h and then 100 *μ*g/mL SGD was added) were cocultured with HEK293 cells. After incubation for 24 h at 37°C, RT-qPCR analysis was used to detect the gene level of TRPV1.

### 2.11. Western Blotting

The colonic tissues were dissected and placed in lysis buffer. The protein concentrations were determined and separated by SDS-PAGE. After transferring to PVDF membranes (Solarbio, China), the blots were incubated with primary antibodies (anti-TRPV1, 1 : 1,000; anti-CAMKII, 1 : 1,000; anti-pCAMKII-T286, 1 : 2,000, Solarbio, China) overnight at 4°C. The membranes were washed three times with PBS, followed by incubation with the corresponding secondary antibody for 1 h. The ECL kit was used to detect the specific bands.

### 2.12. Reverse Transcription-Quantitative PCR (RT-qPCR) Analysis

Total RNA of intestine tissue was extracted from colon tissue using RNA prep Pure Tissue Kit according to the manufacturer's instructions (Tiangen Biotech, China) and reverse-transcribed to cDNA. The primer sequences of TRPV1, calcineurin, TPH, SERT, and *β*-actin are listed in Table 3. The StepOnePlus™ Real-Time PCR System (Applied Biosystems; Thermo Fisher Scientific, Inc.) was used to determine the mRNA levels. Data on mRNA expression were analyzed by the relative amount normalized to that of *β*-actin.

### 2.13. Statistical Analysis

The experimental data are presented as mean ± standard error (SE). One-way ANOVA and Student's *t*-test was used to compare different groups. *P* < 0.05 was considered to indicate statistically significant differences.

## 3. Results

### 3.1. Effects of SGD on TNBS-Induced Visceral Hyperalgesia

To identify the therapeutic effects of SGD on PI-IBS rats, a TNBS-induced PI-IBS rat model was constructed. The DAI scores in PI-IBS rats, determined by body weight, stool consistency, and gross bleeding, were significantly increased by 167% compared with those in the normal group (*P* < 0.001), indicating that the PI-IBS model was successfully established (Figure 2(b)). AWR reflects the pain threshold pressure of the animals. As shown in Figure 2(c), in comparison to the normal rats, the AWR pressure threshold in PI-IBS rats was significantly reduced by 44.3%. Following treatment with SGD, the AWR increased in a dose-dependent manner. The result indicated that SGD exerted an analgesic effect on PI-IBS rats.

### 3.2. Effects of SGD on Histological Changes

**Figure 3:**
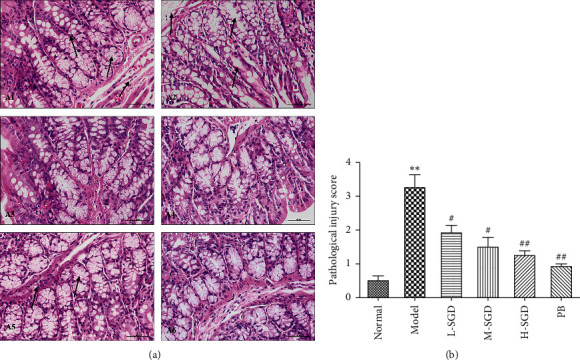
Colon changes in PI-IBS rats under SGD treatment. (a) Colons were stained with H&E for histological grading: (A1) normal group; (A2) PI-IBS group; (A3) L-SGD group; (A4) M-SGD group; (A5) H-SGD group; (A6) PB group. (b) Statistical analysis of pathological injury score. ^*∗∗*^*P* < 0.01 versus normal group; ^#^*P* < 0.05, ^##^*P* < 0.01, and ^###^*P* < 0.001 versus PI-IBS model rats.

Pathological injury scores reflect the histological changes in rats. As shown in Figure 3, the examination of the histological sections confirmed the damage in PI-IBS rats, as exhibited by the difference in tissue architecture between the PI-IBS rats and normal rats. The PI-IBS group displayed pathological changes in the architecture of the colon, such as loss of crypts and epithelial columnar cells, goblet cell depletion, and edema, followed by cell infiltration in the submucosa layer, which resulted in increased pathological injury scores. Following treatment with SGD, the intestinal histological structure was evidently restored, indicating a beneficial effect of SGD on PI-IBS.

### 3.3. Effects of SGD on Colonic EC Cell Number and 5-HT Content in PI-IBS Rats

**Figure 4:**
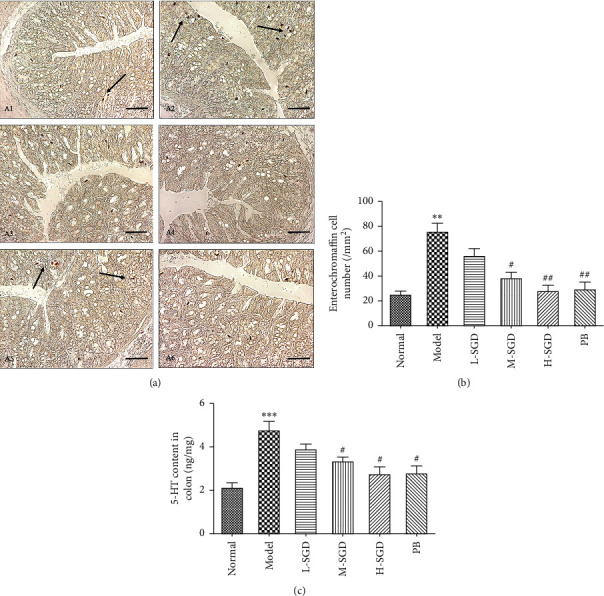
Effects of on SGD enterochromaffin cell number and 5-HT content in colon tissue of rats. (a) Representative enterochromaffin cell immunohistochemical (IHC) staining in rats colon tissue: (A1) normal group; (A2) PI-IBS group; (A3) L-SGD group; (A4) M-SGD group; (A5) H-SGD group; (A6) PB group. Scale bars indicate 50 *μ*m (200x) in each picture. (b) Statistical analysis of enterochromaffin cell number. (c) 5-HT content in colon. Data are presented as mean ± SEM (*n* = 10). ^*∗∗*^*P* < 0.01 versus normal group; ^#^*P* < 0.05 and ^##^*P* < 0.01 versus PI-IBS model rats.

We then sought to determine whether the EC cell number and 5-HT content in the colonic tissue were affected by SGD. As shown in Figures 4(a)–4(c), in comparison to the normal rats, the colonic EC cell numbers in PI-IBS rats were significantly increased from 33 ± 7.1 to 107.7 ± 9.2/mm^2^ (*P* < 0.001), suggesting the occurrence of EC cell hyperplasia in PI-IBS rats. Following SGD treatment, the colonic EC cell numbers were significantly reduced to 45 ± 6.8 per mm^2^ (*P* < 0.05), suggesting that SGD was able to significantly decrease the colonic EC cell density in PI-IBS rats. As the majority of 5-HT in the colon is released from the EC cells, we further analyzed the 5-HT content in the colon. Consistently with the results mentioned previously, the 5-HT content in PI-IBS rats was significantly increased (139.4 ± 15.1%), whereas SGD treatment was able to reverse this increase. Collectively, these results indicated the potential role of the 5-HT signaling pathway in the therapeutic effect of SGD.

### 3.4. Effects of SGD on the mRNA Expression of SERT and TPH in the Colon

**Figure 5:**
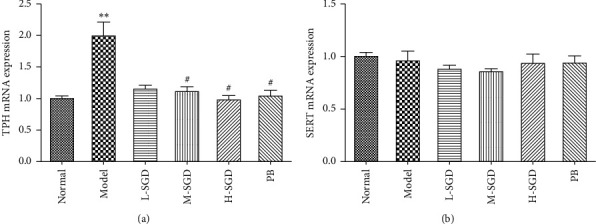
Effects of SGD on TPH and SERT gene mRNA levels in PI-IBS rats. The mRNA levels of (a) TPH and (b) SERT were determined by real-time PCR. Quantitative date of TPH and SERT were standardized against *β*-actin level and expressed as mean ± SEM of three independent experiments. ^*∗∗*^*P* < 0.01 versus normal group; ^#^*P* < 0.05 versus PI-IBS model rats.

To further elucidate whether the role of SGD in alleviating visceral hyperalgesia was associated with the 5-HT signaling pathway, we measured the gene levels of tryptophan hydroxylase (TPH) and serotonin reuptake transporter (SERT). As shown in Figures 5(a) and 5(b), compared with the normal group, the mRNA level of TPH in PI-IBS rats was significantly increased by 98.4%. Following SGD treatment, the mRNA levels of TPH in colonic tissues were decreased by 51.3%. However, there was no significant difference in the SERT gene level between PI-IBS rats in either the control or SGD treatment groups.

### 3.5. Effects of SGD on the Expression and Function of TRPV1 *In Vivo*

**Figure 6:**
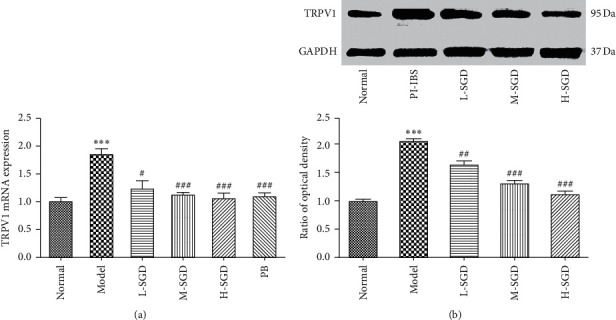
Effects of SGD on TRPV1 mRNA and protein levels in rats. The mRNA levels of TRPV1 were determined by Real-Time PCR (a). The protein levels of TRPV1 were determined by western blot analysis; (b) The results represented as mean ± SEM of three independent experiments. ^*∗*^*P* < 0.05, ^*∗∗*^*P* < 0.01, and ^*∗∗∗*^*P* < 0.001 versus normal group; ^#^*P* < 0.05, ^##^*P* < 0.01, and ^###^*P* < 0.001 versus PI-IBS rats.

**Figure 7:**
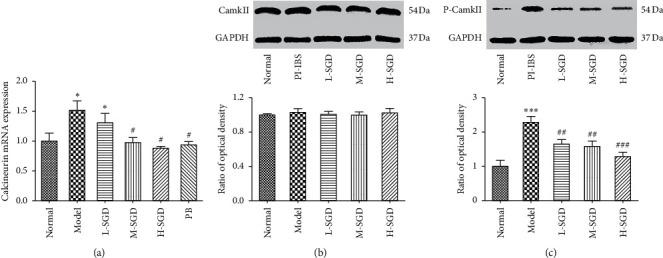
Effects of SGD on the expression of calcineurin, CAMKII, and p-CAMKII in the colon tissue of rats. The mRNA levels of (a) calcineurin were determined by real-time PCR. The protein levels of (b) CAMKII and (c) p-CAMKII were determined by western blot analysis. Data are expressed as mean ± SEM. ^*∗*^*P* < 0.05 and ^*∗∗∗*^*P* < 0.001 versus normal group; ^#^*P* < 0.05, ^##^*P* < 0.01, and ^###^*P* < 0.001 versus PI-IBS rats. CAMKII: Ca^2+^/calmodulin-dependent protein kinase II.

It was reported that TRPV1 plays a key role in visceral hypersensitivity. To determine whether the expression and function of TRPV1 was affected by SGD, the gene and protein expression level of TRPV1 were detect by RT-qPCR and western blot analyses. The expression levels of CAMKII and calcineurin were measured to evaluate the function of TRPV1 [28, 29]. As shown in Figure 6, compared with control rats, the gene and protein expression levels of TRPV1 were significantly increased by 84.6 and 107%, respectively, in PI-IBS rats; however, SGD treatment significantly reversed this increase. Moreover, we also observed that SGD treatment could decrease the expression level of p-CAMKII and calcineurin Figure 7, which are important indices reflecting the function of TRPV1. Collectively, these results indicated that the therapeutic effect of SGD was associated with the expression and function of TRPV1 (Figure 7).

### 3.6. Effects of SGD on the Expression of TRPV1 *In Vitro*

**Figure 8:**
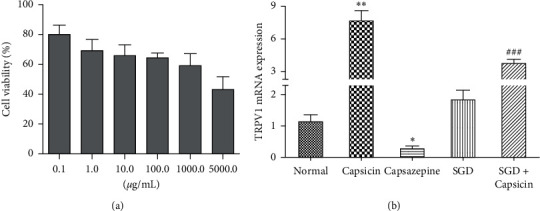
Effects of SGD on the expression of TRPV1 in vitro. (a) CCK-8 cell viability assay of HEK293 cells after treatment with SGD (0.1–5000 *μ*g·ml^−1^) for 24 h. (b) Effects of SGD on gene mRNA levels of TRPV1 in HEK293 cells. ^*∗*^*P* < 0.05 and ^*∗∗*^*P* < 0.01 versus normal group; ^###^*P* < 0.001 versus PI-IBS rats.

To further validate the effect of SGD on TRPV1, another study was performed in HEK293 cells. First, the CCK-8 assay was performed to examine the toxicity of SGD on HEK293 cells. As shown in Figure 8(a), the results demonstrated that SGD exhibited low toxicity against HEK293 cells, and 100 *μ*g·mL^−1^ SGD was selected for further experiments according to the results [30]. Then, capsaicin (TRPV1 agonist) or capsazepine (TRPV1 antagonist) were applied to induce or repress the expression of TRPV1, respectively. As expected, capsaicin strongly induced TRPV1 mRNA and protein levels, whereas capsazepine exerted an obvious inhibitory effect. However, no significant difference was observed in SGD treatment groups compared with the normal group. Following SGD treatment, the gene level of TRPV1 was significantly decreased by 56.7% (Figure 8(b)). These results suggested that SGD may reverse the increased TRPV1 expression.

## 4. Discussion

PI-IBS is a functional gastrointestinal disorder that lacks specific biochemical abnormalities. Traditionally, the clinical treatment of PI-IBS-related symptoms includes antispasmodics, antidepressants, bulking agents and/or laxatives, and opioids [28]. Although these drugs may relieve the symptoms of the patients, they have not achieved satisfactory results due to their associated adverse effects. Thus, it is crucial to identify a new therapeutic approach to relieve the symptoms of visceral pain. TCMs have fewer side effects and exert their therapeutic effects through multiple targets and pathways and have long been applied in the treatment of various diseases [29]. SGD, a TCM prescription, has been widely used to improve the clinical symptoms of abdominal spasmodic pain accompanying acute gastroenteritis. However, the therapeutic mechanism of SGD in PI-IBS-like conditions remains unknown. To the best of our knowledge, the present study was the first to investigate the therapeutic effect of SGD in a TNBS-induced PI-IBS rat model and to examine whether the therapeutic effect of visceral hypersensitivity may be mediated by 5-HT and TRPV1 signaling.

Abnormal bowel habits and visceral hypersensitivity are hallmark clinical manifestations of PI-IBS. However, the mechanisms underlying visceral hypersensitivity in patients with IBS have not been fully elucidated, which has restricted the development of effective pain management strategies. TRPV1 activation was reported to be involved in the hypersensitivity, suggesting its potential as a target of molecular therapies [30]. Moreover, the TRPV1 expression level was found to be significantly increased in nerve fibers of IBS patients [30]. Consistent with these results, the present study demonstrated that the gene and protein levels of TRPV1 were significantly increased in PI-IBS rats and that SGD treatment could reverse this increase. To further evaluate the effect of SGD on TRPV1 expression, HEK293 cells were cocultured with SGD. Our results demonstrated that SGD could reverse the increase in TRPV1 expression. Chronic, low-grade inflammation has been implicated in disease progression and it is involved in the presence of IBS-like symptoms [32]. The plasma levels of proinflammatory cytokines, such as IL-6 and IL-8, were significantly elevated in IBS patients, and these inflammatory factors acted as the agonists of TRPV1 [32]. It has been reported that SGD possesses a multitude of pharmacological activities, including analgesic and anti-inflammatory properties. Moreover, our previous study also demonstrated that SGD inhibited the expression of TNF-*α*, IL-1*β*, IL-6, and IL-18 [15]. Thus, it was inferred that the attenuated visceral hypersensitivity achieved by SGD was mediated by the anti-inflammatory action of SGD via decreased TRPV1 expression. In recent years, the role of TRPV1 in nociception was extensively investigated. It has been reported that the TRPV1 function was altered in visceral hypersensitivity-related diseases, such as gastroesophageal reflux disease, gastric hypersensitivity, and inflammatory bowel disease (IBD) [8]. In addition, the expression levels of the TRPV1 receptor are also increased in IBD. These results indicated that both the expression levels and the function of TRPV1 play key roles in IBD-induced visceral pain. Therefore, the effect of SGD on TRPV1 function was further evaluated. It is well known that TRPV1 must be phosphorylated before being activated, and TRPV1 has multiple phosphorylation sites for PKA, PKC, and CaMKII [33, 34]. CaMKII is a multifunctional enzyme that regulates a number of neuronal systems [34]. The results demonstrated that the gene and protein levels of p-CaMKII were markedly increased in PI-IBS rats, which was consistent with the results in patients with rectal hypersensitivity. The present study also demonstrated that SGD treatment dose-dependently suppressed the protein level of p-CaMKII. In addition, calcineurin activates a Ca^2+^-dependent signal transduction cascade and plays an important role in the effects of TRPV1 [35]. The present study also revealed that the gene level of calcineurin was significantly increased in PI-IBS rats, and SGD treatment reversed this increase. These results suggest that SGD can also suppress the function of TRPV1.

It was reported that tryptophan depletion can cause IBS-related symptoms, such as abdominal pain and altered bowel habits [36, 37]. Drugs that target 5-HT receptors, such as the antagonists ramosetron and alosetron, have been widely used for the treatment of visceral hypersensitivity and rectal sensitivity in IBS patients [37]. This clinical data suggested that 5-HT signaling plays a key role in regulating visceral hypersensitivity and intestinal motility. Consistent with the previous findings, the colonic 5-HT content in PI-IBS rats was found to be significantly increased compared with that in normal rats. SGD treatment significantly decreased the 5-HT level in PI-IBS rats, and this effect was concomitant with attenuated visceral hyperalgesia. Given the vital role of 5-HT in the pathophysiology of PI-IBS, we further tested the alterations of the 5-HT signaling pathway, such as the expression level of TPH and SERT. The findings demonstrated that SGD treatment markedly decreased the gene expression of TPH and the EC cell number. The mechanisms underlying EC cell hyperplasia in PI-IBS have yet to be fully elucidated, but they were considered to be associated with CD4^+^ T lymphocytes, particularly the Th1/Th2 ratio. It was reported that TNF-*α* can downregulate CD4^+^ T-cell response and IL-6 initiates maturation of Th2 cells from Th0; thus, the Th1/Th2 ratio maybe affected by these cytokines [21]. Our previous study demonstrated that SGD treatment could decrease the levels of TNF-*α*, IL-1*β*, IL-6, and IL-18 [13]. Moreover, paeoniflorin, albiflorin, and oxypaeoniflorin, which are considered the main bioactive compounds of PA, were able to inhibit the expression of these inflammatory factors; that is, paeoniflorin reduced the expression of IL-1*β* and TNF-*α*, and albiflorin suppressed the LPS-induced production of NO, IL-6, and TNF-*α*. These results prompted us to hypothesize that the decrease in the 5-HT level induced by SGD may be mediated via decreasing the expression of inflammatory cytokines. It may be inferred that the main reason for the efficacy of SGD in PI-IBS is associated with the active ingredients of each herb, and these ingredients exert therapeutic effects through multiple targets and pathways. To clearly identify the key active ingredients that are responsible for the therapeutic effect, further study is required.

SERT plays an important role in the initiation and termination of 5-HT signaling [36]. In the clinical setting, the antidepressant selective serotonin reuptake inhibitors are widely used for the treatment of IBS. Coates et al. reported a reduced SERT level in IBS patients compared with that in healthy controls [38]. However, the results of Kerckhoffs et al. [39] were the opposite of those reported by Coates et al. The present study demonstrated that there was no significant difference in the expression level of SERT between the PI-IBS and control groups; we also observed that SGD treatment did not affect the expression level of SERT. TPH is the rate-limiting enzyme in 5-HT synthesis, and inhibition of TPH can suppress 5-HT synthesis. TPH is currently considered as a target for the treatment of IBD. Our results indicated that SGD was able to decrease the TPH level, indicating that certain active ingredients in SGD may serve as potent inhibitors of TPH. These results offer a new theoretical basis for including SGD in the treatment of PI-IBS cases that are related to 5-HT signaling. These data may also provide novel insight into clinical applications in the treatment of PI-IBS.

## 5. Conclusion

xIn conclusion, the present study demonstrated that SGD is an effective agent for ameliorating visceral hyperactivity in PI-IBS rats. The therapeutic effect was dose-dependent and 12.5 g^−1^·kg^−1^ d was determined as the optimal dose. We also demonstrated that the regulatory mechanism may involve inactivation of TRPV1 and reduced 5-HT synthesis.

## Figures and Tables

**Table 1:** Disease activity index scoring.

Weight loss rate (%)	Feces consistency	Bleeding	Score
<1	Normal	No bleeding	0
<5	Formed loose stool	Few bloodshot	1
5–10	Loose stool	Slightly bleeding	2
10–15	Diarrhoea	Bleeding	3
>15	Severe diarrhoea	Severe bleeding	4

**Table 2:** Pathological injury scoring.

Crypt damage	Mucosa	Damage extent (%)	Inflammation	Score
None	None	<1	None	0
1/3 damaged	Slight mucosa damage	1–25	Sparse inflammatory infiltrate	1
2/3 damaged	Mild mucosa damage	25–50	Mild inflammatory infiltrate	2
Large area damaged	Mucosa and gland damage	50–75	Inflammatory infiltrate	3
Completely damaged	Severe mucosa damage	75–100	Severe inflammatory infiltrate	4

**Table 3:** List of primers for real-time PCR.

Gene name	Forward primer (5′-3′)	Reverse primer (5′-3′)
TRPV1	TCAACTTCTTCGTCTACTGCTT	CGGTGTTTTTCAGCTTATAGGG
Calcineurin	AGTGAGTGAGTCGTTCCTTAAG	GACACGCTTGAAGCTCTTAAAA
TPH	TCCGTCCTGTGGCTGGTTACC	ACCGTCTCCTCTGAAGCTCCAAG
SERT	GAACTCCTGGAACACTGGCAACTG	TCTGAGCGGCGGCATCTACC
*β*-Actin	AGATTACTGCCCTGGCTCCT	ACATCTGCTGGAAGGTGGAC

## Data Availability

The data used or analyzed during the current study are available from the corresponding author on reasonable request.

## Supplementary Materials

Supplementary Materials First part: the quantity control of SGD. Table S1: the MRM transitions and parameters for the seven analytes in SGD. Table S2: the regression equations of seven analytes in SGD. Table S3: the intrabatch and interbatch precision of seven analytes in SGD. Table S4: stability of seven analytes in SGD. Table S5: the concentrations of the seven analytes in SGD. Second part: serotonin content assessment. Figure S1: product ion mass spectra of 5-HT (a) and typical chromatograms of 5-HT and IS (b). Table S6: the MRM transitions and linearity of 5-HT. Table S7: accuracies, precisions, and stability of 5-HT in rat colon. Figure S2: evaluation of abdominal withdrawal reflex (AWR). Data are presented as mean ± SEM (*n* = 10). ^###^*P* < 0.001 versus PI-IBS model rats.
