# Supplementary material for: Shaoyao-Gancao Decoction Relieves Visceral Hyperalgesia in TNBS-Induced Postinflammatory Irritable Bowel Syndrome via Inactivating Transient Receptor Potential Vanilloid Type 1 and Reducing Serotonin Synthesis
Source: Evid Based Complement Alternat Med. 2020 Oct 15;2020:7830280. doi: 10.1155/2020/7830280 (PMC7584960; doi:10.1155/2020/7830280)
Supplement: Supplementary Materials — First part: the quantity control of SGD. Table S1: the MRM transitions and parameters for the seven analytes in SGD. Table S2: the regression equations of seven analytes in SGD. Table S3: the intrabatch and interbatch precision of seven analytes in SGD. Table S4: stability of seven analytes in SGD. Table S5: the concentrations of the seven analytes in SGD. Second part: serotonin content assessment. Figure S1: product ion mass spectra of 5-HT (a) and typical chromatograms of 5-HT and IS (b). Table S6: the MRM transitions and linearity of 5-HT. Table S7: accuracies, precisions, and stability of 5-HT in rat colon. Figure S2: evaluation of abdominal withdrawal reflex (AWR). Data are presented as mean ± SEM (n = 10). ###P < 0.001 versus PI-IBS model rats. [file 7830280.f1.doc]

**Supplementary materials**

**First part: The quantity control of SGD.**

Supplementary Table 1. The MRM transitions and parameters for the seven analytes in SGD.

| Analyte | MRM transitions (precursor/product) | DP (V) | EP (V) | CE (V) | CXP (V) |
| --- | --- | --- | --- | --- | --- |
| Paeoniflorin | 479.1/121.1 | -120 | -10 | -20 | -10 |
| Albiflorin | 479.1/121.1 | -120 | -10 | -20 | -10 |
| Oxypaeoniflorin | 495.1/137.1 | -150 | -10 | -33 | -10 |
| Liquiritin | 417.3/255.1 | -130 | -10 | -26 | -10 |
| Glycyrrhizin | 821.1/350.7 | -80 | -10 | -58 | -10 |
| Glycyrrhetinic acid | 469.3/355.2 | -122 | -10 | -63 | -10 |
| Liquiritigenin | 255.1/119.0 | -127 | -10 | -37 | -10 |

Supplementary Table 2. The regression equations of seven analytes in SGD.

| Analyte | Regression equation | R^2^ |
| --- | --- | --- |
| Paeoniflorin | y = 0.0018x - 0.0134 | 0.9994 |
| Albiflorin | y = 0.001x - 0.0048 | 0.9993 |
| Oxypaeoniflorin | y = 0.0117x - 0.0188 | 0.9995 |
| Liquiritin | y = 0.0623x + 0.1054 | 0.9999 |
| Glycyrrhizin | y = 0.0024x + 0.0281 | 0.9995 |
| Glycyrrhetinic acid | y = 0.0112x - 0.0828 | 0.9976 |
| Liquiritigenin | y = 0.1996x + 2.9509 | 0.9989 |

Supplementary Table 3. The intra and inter-batch precision of seven analytes in SGD.

| Analyte | Nominal conc. (ng/mL) | Intra-batch | | Inter-batch | |
| --- | --- | --- | --- | --- | --- |
|  |  | Concentration (ng/mL) | RSD(%) | Concentration (ng/mL) | RSD(%) |
| Paeoniflorin | 15.63 | 16.24±1.79 | 5.13 | 16.4±2.32 | 6.60 |
|  | 125 | 126.43±1.89 | 2.38 | 128±0.56 | 3.03 |
|  | 1000 | 1002.46±0.91 | 0.97 | 1018.33±0.25 | 2.26 |
| Albiflorin | 15.63 | 15.45±1.33 | 3.78 | 14.72±1.59 | 7.26 |
|  | 125 | 123.57±2.39 | 2.75 | 121.9±1.23 | 3.26 |
|  | 1000 | 1001.00±0.14 | 1.52 | 1007.6±0.36 | 2.84 |
| Oxypaeoniflorin | 15.63 | 15.96±6.34 | 6.99 | 16.71±2.51 | 8.88 |
|  | 125 | 123.57±2.52 | 2.39 | 129.33±0.62 | 4.29 |
|  | 1000 | 1007.67±0.25 | 0.97 | 1011.2±0.20 | 1.39 |
| Liquiritin | 15.63 | 16.4±3.23 | 6.91 | 14.69±4.10 | 8.33 |
|  | 125 | 120.8±1.49 | 4.33 | 129.69±0.98 | 4.70 |
|  | 1000 | 990.87±0.15 | 1.13 | 1008.07±0.18 | 1.00 |
| Glycyrrhizin | 15.63 | 16.45±2.98 | 7.13 | 16.66±3.38 | 8.03 |
|  | 125 | 128.77±1.22 | 3.90 | 129.43±1.52 | 4.62 |
|  | 1000 | 1011.8±0.13 | 1.37 | 1012.53±0.25 | 1.56 |
| Glycyrrhetinic acid | 15.63 | 16.64±2.03 | 8.23 | 14.32±2.38 | 10.50 |
|  | 125 | 129.75±1.51 | 4.91 | 124.7±0.16 | 3.01 |
|  | 1000 | 992.1±0.28 | 1.01 | 1015.43±00.26 | 1.91 |
| Liquiritigenin | 15.63 | 14.62±3.60 | 8.58 | 16.75±2.43 | 9.12 |
|  | 125 | 122.07±1.49 | 3.22 | 130.73±0.96 | 5.70 |
|  | 1000 | 990.9±0.20 | 1.13 | 1010.33±0.32 | 1.30 |

Supplementary Table 4. Stability of seven analytes in SGD.

| Analyte | Nominal conc.  (ng/mL) | 4 ℃ for 24 h | | -80 ℃ for 21 days | |
| --- | --- | --- | --- | --- | --- |
|  |  | Concentration (ng/mL) | RSD(%) | Concentration (ng/mL) | RSD(%) |
| Paeoniflorin | 15.63 | 14.78±2.38 | 7.00 | 16.9±0.72 | 9.98 |
|  | 125 | 123.02±0.79 | 2.09 | 123.34±0.28 | 1.65 |
|  | 1000 | 992.17±0.19 | 0.98 | 990.83±0.13 | 1.13 |
| Albiflorin | 15.63 | 16.49±2.13 | 7.10 | 14.61±2.44 | 8.29 |
|  | 125 | 128.25±0.50 | 3.22 | 123.1±0.12 | 1.87 |
|  | 1000 | 1010.47±0.23 | 1.30 | 993.1±0.13 | 0.85 |
| Oxypaeoniflorin | 15.63 | 16.76±1.93 | 9.07 | 16.88±1.04 | 9.83 |
|  | 125 | 127.9±1.09 | 3.05 | 128.09±0.16 | 3.03 |
|  | 1000 | 993.17±0.17 | 0.85 | 1008.63±0.09 | 1.06 |
| Liquiritin | 15.63 | 14.28±2.39 | 10.8 | 17.05±1.42 | 11.2 |
|  | 125 | 121.67±0.95 | 3.39 | 122.91±0.72 | 2.17 |
|  | 1000 | 1009.13±0.20 | 1.14 | 992.12±0.20 | 0.98 |
| Glycyrrhizin | 15.63 | 15.01±0.81 | 4.92 | 17.07±1.78 | 9.76 |
|  | 125 | 121.68±0.13 | 2.61 | 128.44±0.29 | 3.38 |
|  | 1000 | 991.83±0.16 | 1.01 | 1009.49±0.05 | 1.16 |
| Glycyrrhetinic acid | 15.63 | 16.51±2.67 | 7.42 | 14.38±3.07 | 10.2 |
|  | 125 | 127.14±0.26 | 2.11 | 123.35±0.42 | 1.67 |
|  | 1000 | 1010.5±0.23 | 1.31 | 992.4±0.15 | 0.94 |
| Liquiritigenin | 15.63 | 14.77±1.48 | 6.88 | 17.08±1.37 | 11.4 |
|  | 125 | 127.44±0.23 | 2.41 | 128.25±0.33 | 3.20 |
|  | 1000 | 1010.53±0.15 | 1.30 | 1011.62±0.09 | 1.43 |

Supplementary Table 5. The concentrations of the seven analytes in SGD.

| Analyte | Concentration (mg/mL) |
| --- | --- |
| Paeoniflorin | 16.8±2.31 |
| Albiflorin | 21.4±1.85 |
| Oxypaeoniflorin | 13.6±1.14 |
| Liquiritin | 18.2±2.46 |
| Glycyrrhizin | 20.7±3.88 |
| Glycyrrhetinic acid | 7.05±0.92 |
| Liquiritigenin | 9.01±1.75 |

**Second part: Serotonin content assessment**

Supplementary Table 6. The MRM transitions and linearity of 5-HT.

| Analyte | MRM transitions (precursor/product) | Parameter | | | | | Linearity | |
| --- | --- | --- | --- | --- | --- | --- | --- | --- |
|  |  | DP (V) | | EP (V) | CE (V) | CXP (V) | Regression equation | R^2^ |
| 5-HT | 385.2/177.1 | 130 | 10 | | 17 | 16 | y = 0.0006x + 0.0193 | 0.9992 |

Supplementary Table 7. Accuracies, precisions, and stability of 5-HT in rat colon.

| Analyte | Nominal conc. (ng/mL) | Intra-batch | | Inter-batch | | Stability | |
| --- | --- | --- | --- | --- | --- | --- | --- |
|  |  | Accuracy (%) | Precision (%) | Accuracy (%) | Precision (%) | 4 ℃ for 24 h  RSD (%) | -80 ℃ for 21 days  RSD (%) |
| 5-HT | 31.25 | 1.46 | 2.04 | -0.19 | 1.23 | 1.11 | 0.96 |
|  | 250 | 4.31 | 2.42 | 1.83 | 1.85 | 3.24 | 2.39 |
|  | 1000 | 6.08 | 5.29 | 7.49 | 9.31 | 5.33 | 6.79 |

Supplementary Figure 1. Product ion mass spectra of 5-HT (A) and typical chromatograms of 5-HT and IS (B).

Supplementary Figure 2. Evaluation of disease activity index (DAI). Data are presented as mean ± SEM (n=10). ###P <0.001 versus PI-IBS model rats.
